# Supplementary material for: Climate effects of ecosystem change converge according to the ratio of the daytime to daily vapor flux
Source: Innovation (Camb). 2025 Jan 6;6(1):100733. doi: 10.1016/j.xinn.2024.100733 (PMC11763916; doi:10.1016/j.xinn.2024.100733)
Supplement: Document S1. Figure S1–S7 and Table S1 [file mmc1.pdf]

The Innovation, Volume *6*

Supplemental Information

Climate effects of ecosystem change converge according to the ratio of the daytime to daily vapor flux

Langqin Hua, Lin Li, Wenjing Chen, Xuemeng Wang, Xin Xiong, and Guoyi Zhou

Supplemental Information

Climate effects of ecosystem change converge according to the ratio of daytime

to daily vapor flux

DOI:

Langqin Hua, Lin Li, Wenjing Chen, Xuemeng Wang, Xin Xiong, and Guoyi Zhou

Table of Contents

Figures S1 to S7

Tables S1

REFERENCES

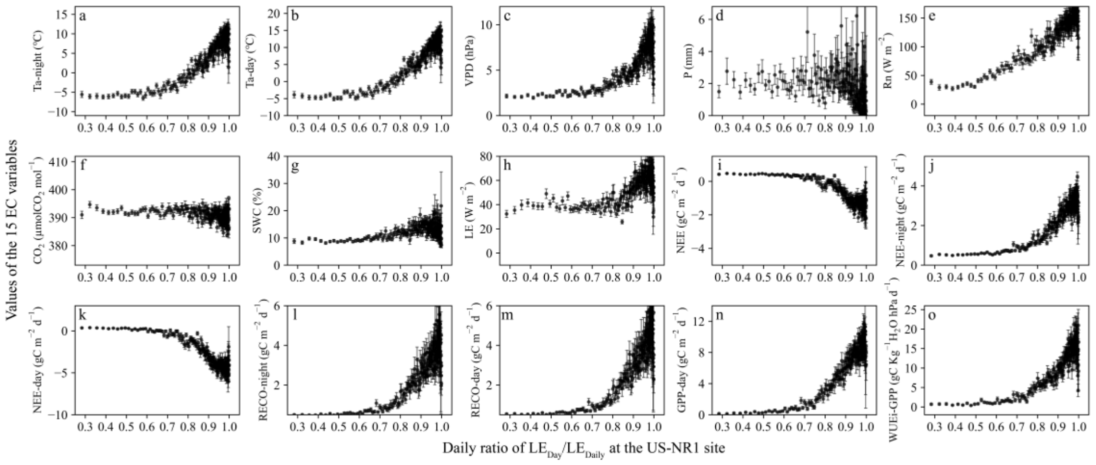

Figure S1. Changes in eddy-covariance variables with the daily ratio of the daytime to daily vapor flux (the daily RATIO) at the US-NR1 site. The variables include a) Ta-night - air temperature in the non-photosynthetic period, b) Ta-day - air temperature in the photosynthetic period, c) VPD - vapor pressure deficit, d) P - daily precipitation, e) Rn - net radiation, f) CO_2 - atmospheric CO_2 concentration, g) SWC - soil water content, h) LE - vapor flux (evapotranspiration), i) NEE - net carbon exchange of ecosystem, j) NEE-night - net carbon exchange of ecosystem at night, k) NEE-day - net carbon exchange of ecosystem in daytime, l) RECO-night - ecosystem respiration at night, m) RECO-day - ecosystem respiration in daytime, n) GPP-day - gross primary production in daytime, and o) WUEi-GPP - inherent water use efficiency (equal to GPP-day*VPD/LE-day). See Pastorello et al.[1] for more detailed information about the site and variables.

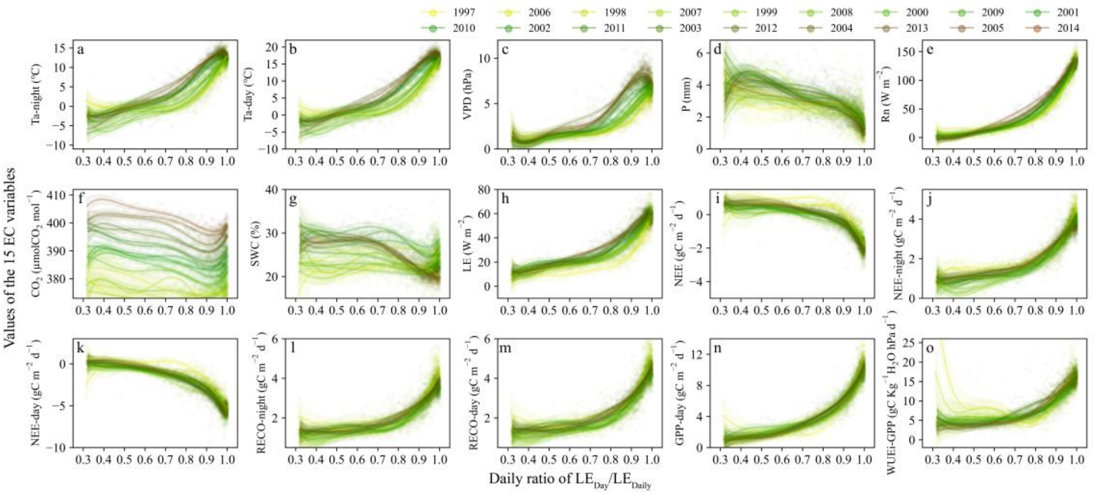

Figure S2. Variations of eddy covariance variables with the daily ratio of the daytime to daily vapor flux (the daily RATIO) for each year during 1997-2014. The fitted curves are all statistically significant (p<0.05). The variables include a) Ta-night - air temperature in the non-photosynthetic period, b) Ta-day - air temperature in the photosynthetic period, c) VPD - vapor pressure deficit, d) P - daily precipitation, e) Rn - net radiation, f) CO_2 - atmospheric CO_2 concentration, g) SWC - soil water content, h) LE - vapor flux (evapotranspiration), i) NEE - net carbon exchange of ecosystem, j) NEE-night - net carbon exchange of ecosystem at night, k) NEE-day - net carbon exchange of ecosystem in daytime, l) RECO-night - ecosystem respiration at night, m) RECO-day - ecosystem respiration in daytime, n) GPP-day - gross primary production in daytime, and o) WUEi-GPP - inherent water use efficiency (equal to GPP-day*VPD/LE-day). See Pastorello et al.[1] for more detailed information about the site and variables.

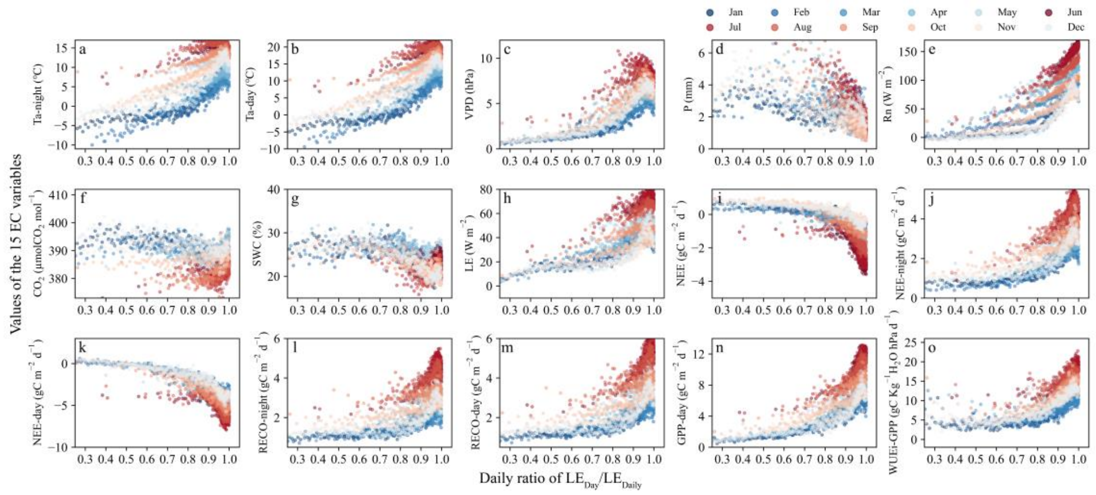

Figure S3. Monthly eddy covariance variables with the daily ratio of the daytime to daily vapor flux (the daily RATIO) during 1997-2014. The variables include a) Ta-night - air temperature in the non-photosynthetic period, b) Ta-day - air temperature in the photosynthetic period, c) VPD - vapor pressure deficit, d) P - daily precipitation, e) Rn - net radiation, f) CO_2 - atmospheric CO_2 concentration, g) SWC - soil water content, h) LE - vapor flux (evapotranspiration), i) NEE - net carbon exchange of ecosystem, j) NEE-night - net carbon exchange of ecosystem at night, k) NEE-day - net carbon exchange of ecosystem in daytime, l) RECO-night - ecosystem respiration at night, m) RECO-day - ecosystem respiration in daytime, n) GPP-day - gross primary production in daytime, and o) WUEi-GPP - inherent water use efficiency (equal to GPP-day*VPD/LE-day). See Pastorello et al.[1] for more detailed information about the site and variables.

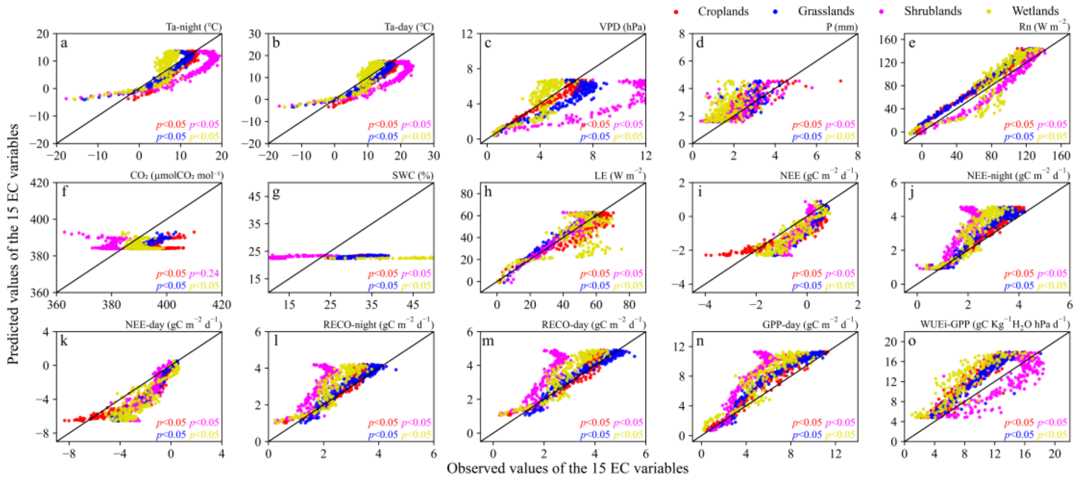

Figure S4. Comparison of predicted and observed daily values of the 15 variables for cropland, grassland, shrubland and wetland. The observed values are the measurements in the four land cover types. The predicted values are based on the measured daily RATIOs in the four land cover types and the regressed relationship between EC variables and daily RATIO in forest. *p* values indicate the significance of the relationships between predicted and observed values. Shrubland in the figure is the combination of shrubland and savanna in Pastorello et al.[1]

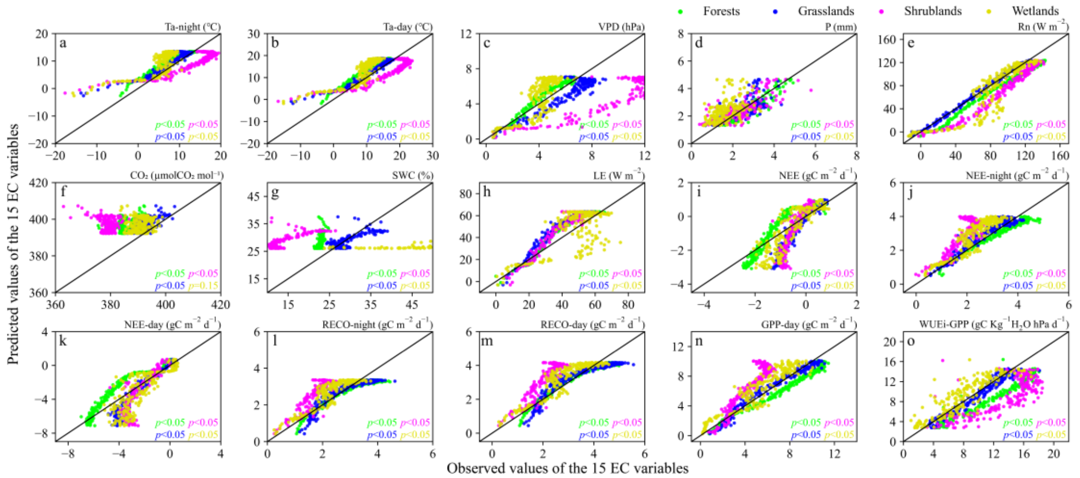

Figure S5. Comparison of predicted and observed daily values of the 15 variables for forest, grassland, shrubland and wetland. The observed values are the measurements in the four land cover types. The predicted values are based on the measured daily RATIOs in the four land cover types and the regressed relationship between EC variables and daily RATIO in croplands. *p* values indicate the significance of the relationships between predicted and observed values. Shrubland in the figure is the combination of shrubland and savanna in Pastorello et al.[1]

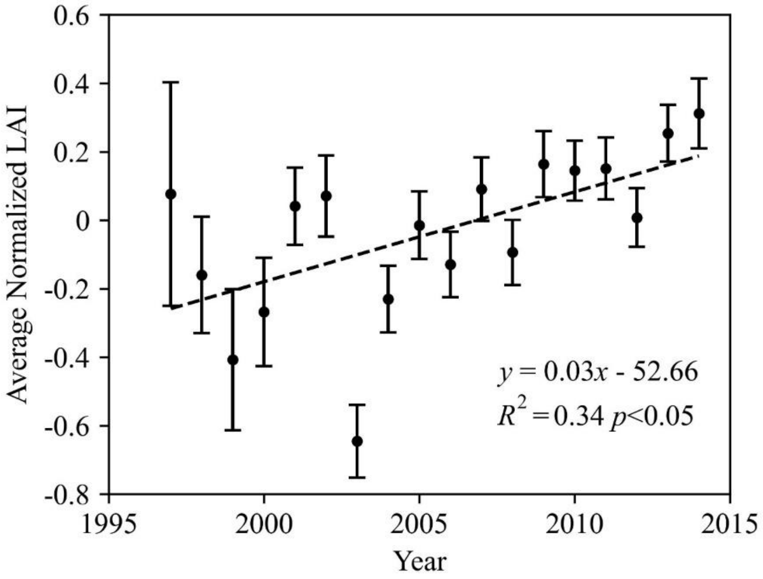

Figure S6. The tendency of global leaf area index (LAI) from 1997-2014. The LAI data were standardized using z-score normalization for each site. Error bars indicate the standard error of the mean.

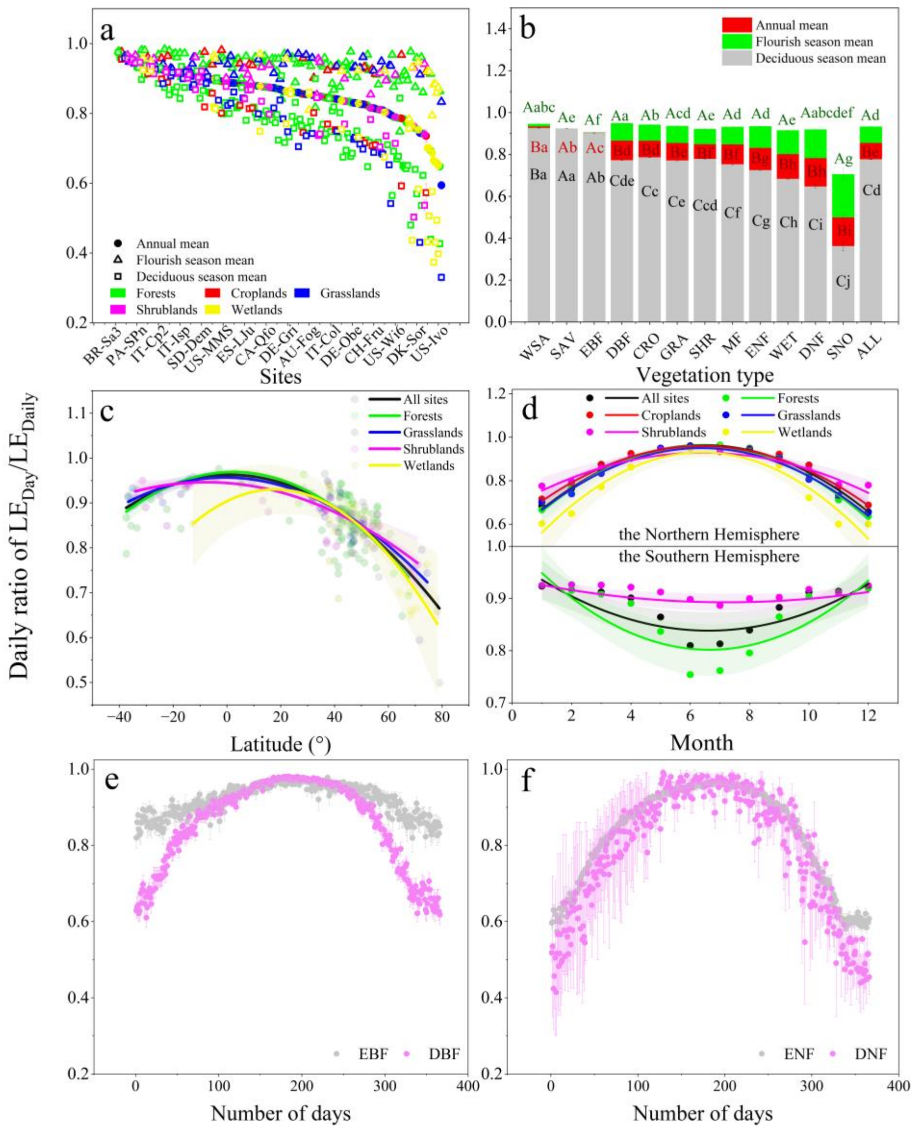

Figure S7. Daily ratio of the daytime to daily vapor flux (the daily RATIO). Averaged according to **a**) eddy covariance (EC) sites, **b**) vegetation types, **c**) latitudes and **d**) months. In-year process of daily RATIO for **e**) evergreen broadleaved forest (grey color) and deciduous broadleaved forest (pink color), **f**) evergreen needle-leaved forest (grey color) and deciduous needle-leaved forest (pink color). BR-Sa3, …, NO-Blv - names of the 212 sites, WSA - woody savanna, SAV - savanna, EBF - evergreen broadleaved forest, DBF - deciduous broadleaved forest, CRO - cropland, GRA - grassland, SHR - shrubland, MF - mixed forest, ENF - evergreen needle-leaved forest, WET - wetlands, DNF - deciduous needle-leaved forest, SNO - snow and ice cover.

Error bars indicate standard errors of the means. Different capital letters indicate a significant ($p<0.05$) difference between annual, flourish season and deciduous season mean daily RATIO, while the different lowercase letters indicate a significant ($p<0.05$) difference between different vegetation types. Flourish season refers to April to September in the Northern Hemisphere and October to March of the following year in the Southern Hemisphere, while deciduous season refers to the other six months of the year.

Table S1 Contributions of the nine significant contributors to daily RATIO in accordance with climate regions and ecosystem types

	All	Tropic	Subtropics	Temperate	Frigid	Forest	Crop	Grass	Shrubsav	Wet
R^2	0.62	0.59	0.52	0.60	0.85	0.63	0.63	0.66	0.55	0.65
n	258180	24568	54166	173828	5618	142980	26444	50603	37165	988
LAI	6.49	22.25	8.16	6.56	2.14	6.51	5.95	6.35	10.31	16.61
ΔT	11.20	17.49	21.76	12.49	2.93	9.28	17.54	13.93	17.09	20.62
Day Length	43.73	2.73	10.64	44.61	81.12	45.03	42.05	47.65	26.36	4.13
P	4.51	5.43	6.92	4.86	0.96	4.77	4.57	3.16	5.11	2.45
SWC	6.05	15.94	9.18	6.18	2.42	5.77	6.08	6.51	9.03	12.38
CO_2	5.73	8.17	8.64	5.87	3.35	5.67	5.76	5.39	7.47	6.30
P/PET	5.94	6.80	7.36	6.07	1.82	5.42	6.09	4.87	5.69	10.94
VPD	9.32	8.84	16.38	6.25	3.09	9.80	6.46	5.93	9.67	19.90
WS	7.03	12.35	10.96	7.11	2.17	7.75	5.50	6.21	9.27	6.67

Note: LAI - leaf area index, ΔT - difference in temperature between day and night, Day Length, P - daily precipitation, SWC - soil water content, CO_2 - atmospheric CO_2 concentration, P/PET - wetness index (P - annual precipitation, PET - annual potential evapotranspiration), VPD - vapor pressure deficit, WS - wind speed. Forest - forest land, Crop - cropland, Grass - grassland, Shrubsav - collection of shrubland, savanna and woody savanna, Wet - wetland.

REFERENCES

1. Pastorello, G., Trotta, C., Canfora, E., et al. (2020). The FLUXNET2015 dataset and the ONEFlux processing pipeline for eddy covariance data. Sci. Data ***7***(1): 225. DOI: https://doi.org/10.1038/s41597-020-0534-3.
