# Supplementary material for: Climate effects of ecosystem change converge according to the ratio of the daytime to daily vapor flux
Source: Innovation (Camb). 2025 Jan 6;6(1):100733. doi: 10.1016/j.xinn.2024.100733 (PMC11763916; doi:10.1016/j.xinn.2024.100733)
Supplement: Document S2. Article plus supplemental information [file mmc2.pdf]

OPEN ACCESS

Climate effects of ecosystem change converge according to the ratio of the daytime to daily vapor flux

Langqin Hua,[1,3] Lin Li,[1,3] Wenjing Chen,[1,3] Xuemeng Wang,[1] Xin Xiong,[2] and Guoyi Zhou[1,*]

*Correspondence: gyzhou@nuist.edu.cn

Received: April 22, 2024; Accepted: November 11, 2024; Published Online: January 6, 2025; https://doi.org/10.1016/j.xinn.2024.100733

© 2024 The Author(s). Published by Elsevier Inc. on behalf of Youth Innovation Co., Ltd. This is an open access article under the CC BY license (http://creativecommons.org/licenses/by/4.0/).

GRAPHICAL ABSTRACT

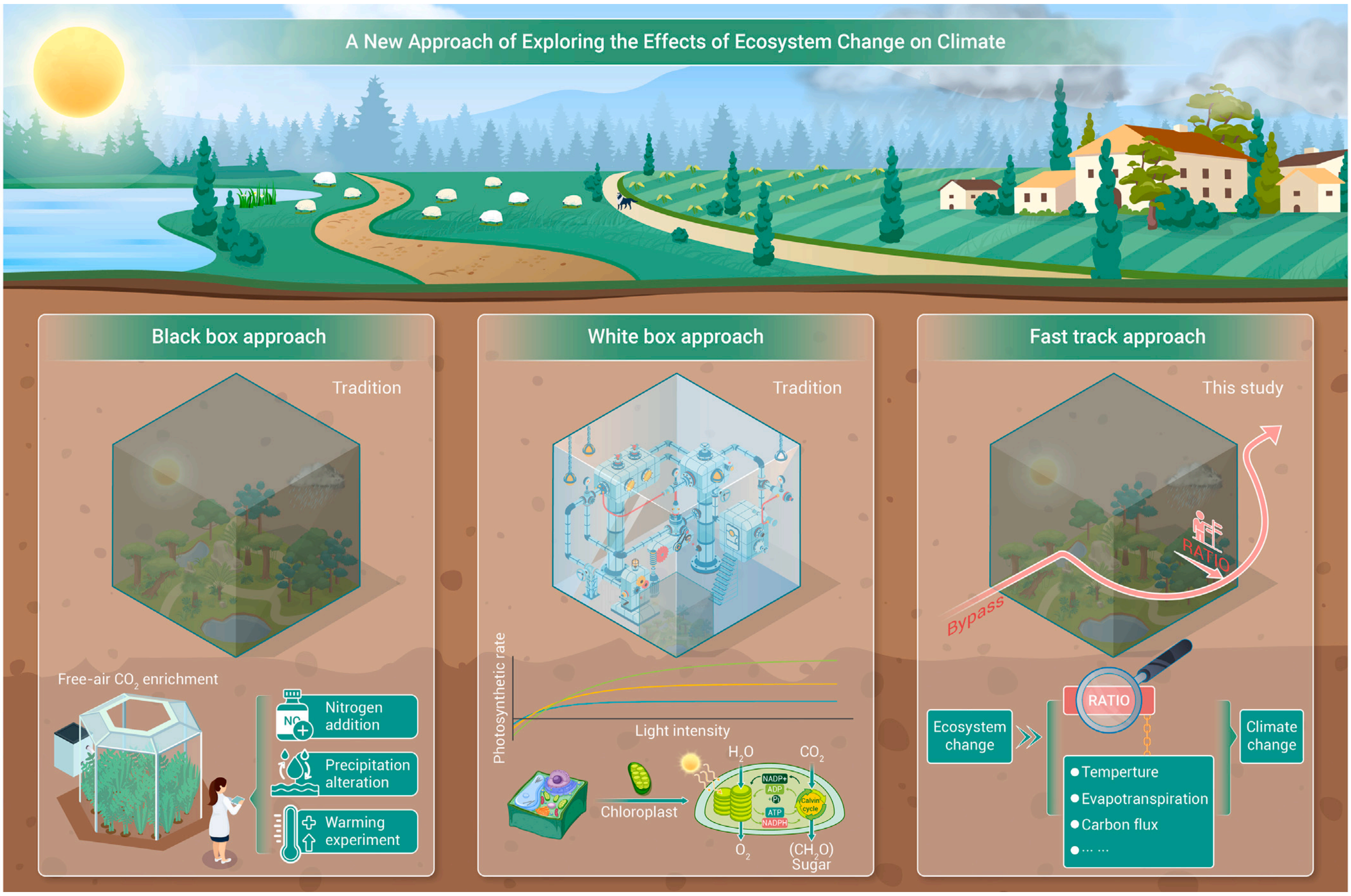

PUBLIC SUMMARY

- A new approach for exploring the effects of ecosystem changes on climate.
- The RATIO can be used to predict climate-related effects under ecosystem changes.
- Eddy covariance observations in an ecosystem or place can be well mapped to other ecosystems or places in accordance with their daily RATIO values.
- Compared with evapotranspiration, the daily RATIO is a better indicator of the effects of ecosystem changes on the land surface temperature.

OPEN ACCESS

REPORT

Climate effects of ecosystem change converge according to the ratio of the daytime to daily vapor flux

Langqin Hua,[1,3] Lin Li,[1,3] Wenjing Chen,[1,3] Xuemeng Wang,[1] Xin Xiong,[2] and Guoyi Zhou[1,*]

[1]Institute of Ecology, School of Ecology and Applied Meteorology, Nanjing University of Information Science & Technology, Nanjing 210044, China

[2]Jiangxi Provincial Key Laboratory of Carbon Neutrality and Ecosystem Carbon Sink, Lushan Botanical Garden, Jiangxi Province and Chinese Academy of Sciences, Jiujiang 332900, China

[3]These authors contributed equally

*Correspondence: gyzhou@nuist.edu.cn

Received: April 22, 2024; Accepted: November 11, 2024; Published Online: January 6, 2025; https://doi.org/10.1016/j.xinn.2024.100733

© 2024 The Author(s). Published by Elsevier Inc. on behalf of Youth Innovation Co., Ltd. This is an open access article under the CC BY license (http://creativecommons.org/licenses/by/4.0/).

Citation: Hua L., Li L., Chen W., et al., (2024). Climate effects of ecosystem change converge according to the ratio of the daytime to daily vapor flux. The Innovation **6(1)**, 100733.

Ecosystem changes can simultaneously generate various climate-related effects, such as evapotranspiration (vapor flux) effects, carbon-cycle effects, and surface temperature effects. These effects are coupled with one another because they are generated through the same biophysical and biogeochemical processes. Consequently, given an easily measurable effect, other effects can be predicted from the measured effect. Here, based on global eddy covariance (EC) observations, we show that the ratio of the daytime to daily vapor flux (RATIO) reflects the complexity of various ecosystem types and is highly coupled with climate effects of ecosystem changes. For the same daily RATIO, the magnitudes of the same EC variable remain unchanged across all of the ecosystems and, thus, EC observations for an ecosystem or place can be mapped to other ecosystems or places in accordance with their daily RATIO values. By applying the daily RATIO, the effects of ecosystem changes on the surface temperature in different climatic zones (including the Tibetan Plateau) can be predicted, which is highly consistent with all previous studies. We found that cooling or warming effects are controlled by the RATIO, not by enhanced or reduced evapotranspiration as many studies have suggested. This study provides a new and simple approach for evaluating the climate effects of ecosystem changes at all spatial-temporal scales worldwide.

INTRODUCTION

Ecosystem changes have complex impacts on Earth's climate through the interlinked biophysical and biogeochemical (BAB) feedbacks.[1-5] Land surface models combined with remote sensing have been used to simulate such feedback and to study the impacts of ecosystem changes on past and future climates.[6,7] Such studies have reached many meaningful conclusions about the effects of vegetation changes on climate, especially on the surface air temperature, in boreal, temperate, and tropical zones.[8,9] Regarding the pathway from ecosystem changes to climate effects, a series of BAB feedback chains and even feedback nets have been established and it has been found that the final climate effects are actually the results of sufficient feedback from many interlinked BAB processes. Consequently, such numerous feedbacks are not well understood.[10] The sign and magnitude of climate changes also depend on the specific ecosystem transitions, their timing, and their location, as well as on the background climate.[6] Vegetation features or more broadly, ecosystem features, such as plant functional traits,[11] leaf area index (LAI),[12] vegetation structure,[13] albedo,[8,14,15] soil moisture, soil organic carbon,[16,17] and background climate,[13,18,19] have been shown to exert significant influences on the climate effects of ecosystem changes. This means that the feedback between vegetation changes and climate is not only complex but is also unique for each land use type and each time. The capacity of land surface models to accurately reproduce the interplay between vegetation and climate across the globe remains unclear.[6,14]

In this study, we used a different approach to study the impact of ecosystem changes on climate. An ecosystem produces product A and products X_i ($i = 1, 2, \ldots$), where product A can be easily measured. Although A and X_i may be different, they are all produced through the same interlinked BAB processes, somewhat analogous to the different products of the same chemical reaction and, thus, the signs and magnitudes of A and X_i should be coupled. The responses of X_i to ecosystem changes will be mirrored by the effects of the ecosystem changes on A. Under this principle, in this study, we investigated how products X_i are coupled with product A, instead of focusing on how ecosystem changes impact products X_i, which previously has been done in model simulations. The ratio of the daytime to daily evapotranspiration (vapor flux) (RATIO) is taken as product A, and climate effects are taken as products X_i. Ecosystem evapotranspiration depends on the same interlinked BAB processes as climate effects[20,21] and has been proven to be highly coupled with energy fluxes and carbon uptake.[22-24] In addition, it is easily measurable through eddy covariance (EC) observations and remote sensing.

RESULTS AND DISCUSSION

Coupling of EC variables and the daily RATIO

The couplings between 15 EC variables and the daily RATIO for different vegetation types and timescales are considered. Figures 1 and S1–S3 show the couplings for different vegetation types, a single site, and on the annual and monthly scales, respectively. The 15 EC variables can be classified into three types, i.e., eight flux variables (LE, NEE, NEE-night, NEE-day, RECO-day, RECO-night, GPP-day, and WUEi-GPP), four non-flux and energy variables (referred to as energy variables) (Ta-day, Ta-night, Rn, and VPD), and three non-flux and matter variables (referred to as matter variables) (P, CO_2, and SWC).

The change patterns (curves) of the EC variables in relation to the daily RATIO are highly coordinated and consistent between ecosystems (Figures 1 and S1) and between timescales (Figures S2 and S3). All of the tight coupling relationships can be well simulated using random forest model or polynomial model. The 12 flux and energy variables increase (decrease for NEE and NEE-day) nonlinearly as the daily RATIO increases when the daily RATIO is <0.93–0.95, and they decrease when the daily RATIO is >0.93–0.95. The three matter variables decrease in an approximately linear manner when the daily RATIO is <0.93–0.95. However, although the change patterns are significant for all 15 EC variables, the significance is highest for the flux variables, moderate for the energy variables, and lowest for the matter variables.

For the same daily RATIO, the magnitudes of the same EC variable among different ecosystems or sites are quite approximate (Figures 1 and S1). This can be expressed as follows:

$$\text{EC variable}_{i,j}\big|_{\text{daily RATIO} = x} \approx \text{EC variable}_{i,k}\big|_{\text{daily RATIO} = x} \quad x \in (0,1), \qquad \text{(Equation 1)}$$

where, i denotes any one of the 15 EC variables (Ta-night, Ta-day, …, WUEi-GPP), j and k denote any two ecosystems, sites, or ecosystem changes; and x changes within the range of 0–1. The discrepancy in the magnitudes of the same EC variable between ecosystems is smallest for the flux variables, moderate for the energy variables, and greatest for the matter variables. For the same daily RATIO, the magnitudes of the same flux variable between ecosystems are nearly equal when the daily RATIO is <0.7.

The interlinked BAB processes in different ecosystems (or lands), such as evapotranspiration, carbon assimilation, and respiration, are analogous to the coupled chemical reaction processes in different media, while energetic and biological environments such as the air temperature, net radiation, vapor pressure deficit, and microorganisms are the physical and biological catalysts or inhibitors. The quantitative relationship between the different substances directly involved in BAB processes is determined by the BAB processes, regardless of the ecosystem type, which provides the theoretical base for the finding that any two ecosystems exert the same climate effects if their daily RATIOs are similar. In real ecosystems, quantifying these relationships is highly challenging because all of the types of matter are present together and their sources and sinks are intertwined. The more complex the ecosystems are and the longer the timescale

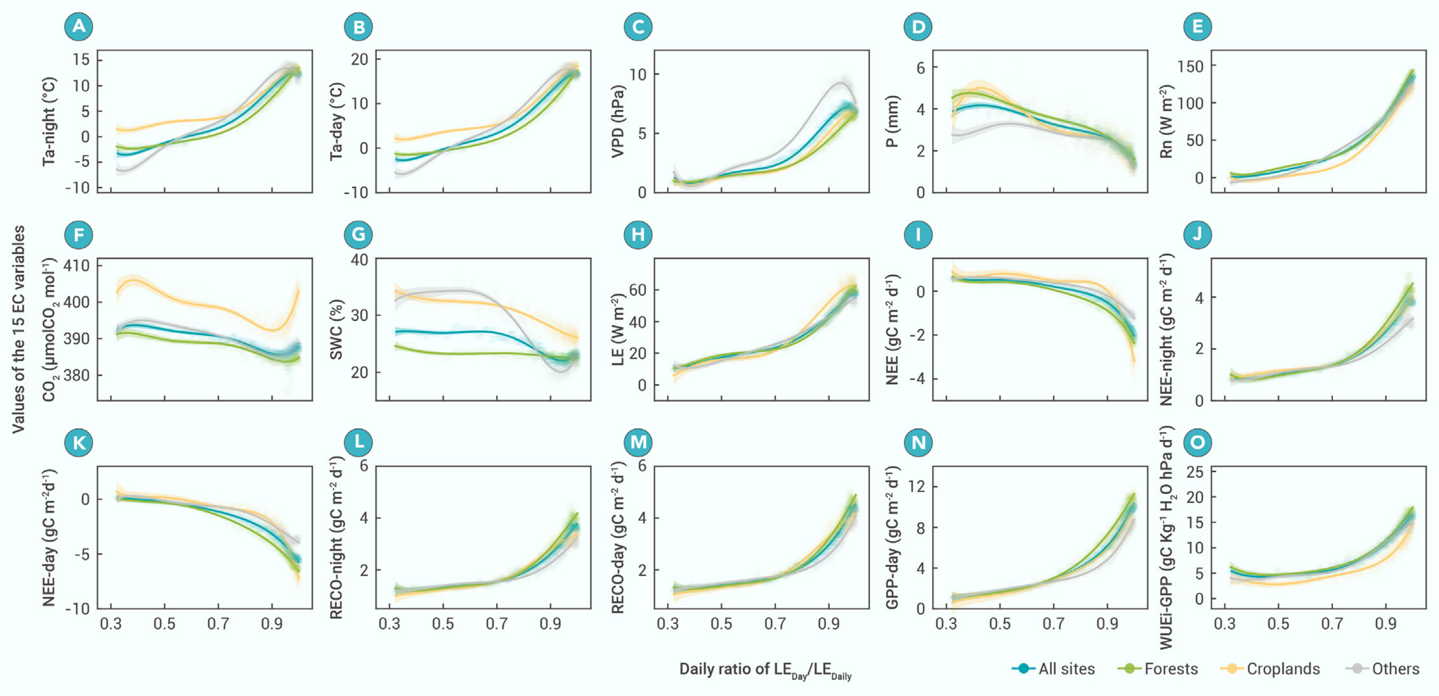

Figure 1. Changes in eddy covariance variables with the daily RATIO for the 18 years during 1997–2014 For clarity, only four groups of ecosystems (all sites, forest sites, cropland sites, and other (shrubland, savannas, grassland, and wetland)) are shown. The fitted curves are all statistically significant ($p < 0.05$). The variables include (A) Ta-night (air temperature in the non-photosynthetic period), (B) Ta-day (air temperature in the photosynthetic period), (C) VPD (vapor pressure deficit), (D) P (daily precipitation), (E) Rn (net radiation), (F) CO_2 (atmospheric CO_2 concentration), (G) SWC (soil water content), (H) LE (vapor flux [evapotranspiration]), (I) NEE (net carbon exchange of ecosystem), (J) NEE-night (net carbon exchange of ecosystem at night), (K) NEE-day (net carbon exchange of ecosystem in daytime), (L) RECO-night (ecosystem respiration at night), (M) RECO-day (ecosystem respiration in daytime), (N) GPP-day (gross primary production in daytime), and (O) WUEi-GPP (inherent water use efficiency [equal to GPP-day × VPD/LE-day]). For more detailed information about the variables' units, see Pastorello et al.[25]

studied is, the more BAB processes and interfaces on which BAB processes occur are included, and thus the less accurate the quantification will be. The very tight coupling relationships between the eight flux variables and the daily RATIO (Figures 1 and S1–S3) demonstrate that EC observations accurately reflect the magnitudes of the substrates or products involved in the BAB processes; otherwise, such consistent and tight relationships would not likely exist. In addition, the change in the daily RATIO from 0 to 1 also corresponds to the increase in the ecosystem complexity; as a result, the difference in the magnitudes of the same flux variable in different ecosystems gradually increases due to a decrease in the accuracy of the quantification. The relationships between the three matter variables and the daily RATIO are the least tight because only part of their measured values join the coupled BAB processes. The other four energy variables act as catalysts and have moderately tight relationships with the daily RATIO because each of them functions as an indivisible whole when catalyzing the BAB processes.

In summary, the daily RATIO reflects the ecosystem complexity and is tightly coupled with the EC variables across various ecosystems, sites, and timescales. For a given daily RATIO, the magnitudes of the same EC variable remain relatively unchanged across various ecosystems and sites. The two properties of the daily RATIO are most apparent for the flux variables, followed by the energy variables, and are the least apparent for the matter variables.

Using the daily RATIO to map EC observations from one place to other places

Because of the two properties of the daily RATIO, we can use the quantified couplings between the daily values of the EC variables and the daily RATIO established in a specific place or ecosystem to estimate the daily values of the same variables in other regions or ecosystems. Figure 2 shows the estimated results for the Eastern Hemisphere, which are based on the observed daily RATIOs in the Eastern Hemisphere and the quantified couplings between the daily values of the EC variables and the daily RATIOs established in the Western Hemisphere. The estimated magnitudes for the 12 flux and energy variables agree well with the observed values. Although some estimated and observed values do not plot close to the 1:1 line, they are significantly correlated with each other ($p < 0.05$). Similarly, we investigated the suitability of using EC data for one ecosystem to estimate the daily changes in the variables in different ecosystems. Figures S4 and S5 show the estimated daily values of EC variables for other land cover types based on daily EC observations for forest and cropland sites, respectively. The estimations are acceptable, except for the three matter variables (P, CO_2, and SWC), which is the same situation shown in Figure 2.

Temporal trends of EC variables for different daily RATIOs

Figure 3 illustrates how the temporal trends (expressed as the linear correlation coefficient r, $r = \pm\sqrt{R^2}$) of the 15 variables change with the daily RATIO during 1997–2014.

Although several variations exist, the temporal trends of all 15 variables vary regularly with the daily RATIO, regardless of whether they are flux, energy, or matter variables.

The temporal trends of the eight carbon-cycle-related variables (CO_2 concentration, NEE, NEE-night, NEE-day, RECO-night, RECO-day, GPP-day, and WUEi-GPP) for different daily RATIOs exhibit high synergic changes (Figures 3F and 3I–3O). The temporal trend of the CO_2 concentration with the daily RATIO is linear, and the r value is close to 1. The CO_2 concentrations increase significantly ($r > 0.47$) for all of the daily RATIOs (all ecosystems), which mirrors the reality of the atmospheric CO_2. For daily RATIOs of 0.75–0.95, the r values are lower where the carbon sequestration and water use efficiency function efficiently, temporal trends of the NEE and NEE-day significantly decrease ($r < -0.47$), and those of the GPP-day and WUEi-GPP significantly increase ($r > 0.47$). Outside of this daily RATIO range, the temporal trends of the NEE, NEE-day, GPP-day, and WUEi-GPP become non-significant ($-0.47 < r < 0.47$).

The temporal trends of the three water-cycle-related variables (P, SWC, and LE) at different daily RATIOs are highly complementary (Figures 3D, 3G, and 3H). The complementarity is constrained by the equation $P = SWC + LE + R$ (R-runoff).

The temporal trends of the four energy-related variables (Ta-night, Ta-day, VPD, and Rn) at different daily RATIOs are highly consistent (Figures 3A–3C and 3E). Here, we take Ta-day as an example to show in detail how the temporal trend of Ta-day changes with the daily RATIO. As shown in Figure 3B, Ta-day decreases ($r < 0$) and increases ($r > 0$) with the daily RATIOs of <0.55 and >0.55, respectively. Ecosystems with a daily RATIO of <0.55 have a strong cooling effect, which offsets global warming. However, whether ecosystems with daily

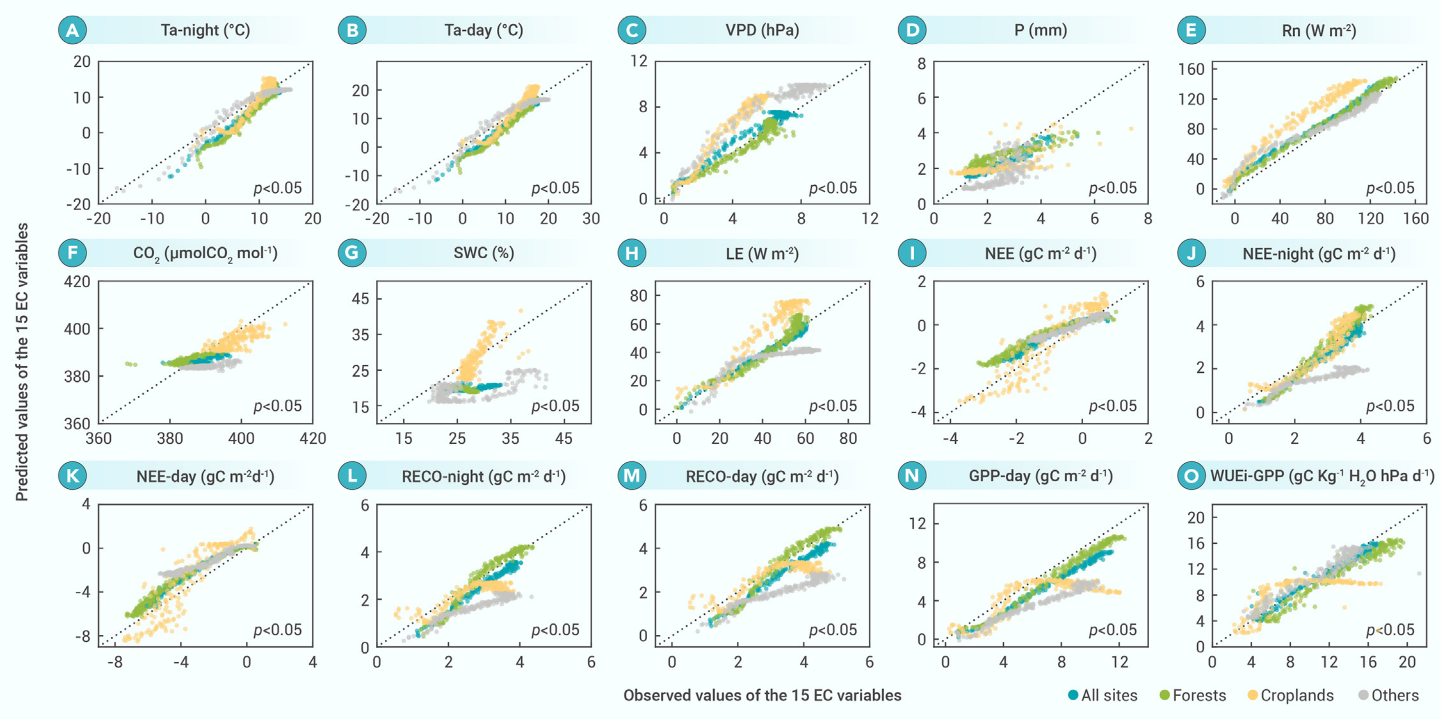

Figure 2. Comparison of predicted and observed daily values of the 15 variables in the Eastern Hemisphere The observed values are the measurements in the Eastern Hemisphere. The predicted values are based on the measured daily RATIOs for the Eastern Hemisphere and the coupling relationship between the EC variables and the daily RATIO in the Western Hemisphere. The *p* values indicate the significance of the relationships between the predicted and observed values for the four land cover types (all sites, cropland, forest, and others [shrubland, savanna, grassland, and wetland]). (A) Ta-night (air temperature in the non-photosynthetic period), (B) Ta-day (air temperature in the photosynthetic period), (C) VPD (vapor pressure deficit), (D) P (daily precipitation), (E) Rn (net radiation), (F) CO_2 (atmospheric CO_2 concentration), (G) SWC (soil water content), (H) LE (vapor flux [evapotranspiration]), (I) NEE (net carbon exchange of ecosystem), (J) NEE-night (net carbon exchange of ecosystem at night), (K) NEE-day (net carbon exchange of ecosystem in daytime), (L) RECO-night (ecosystem respiration at night), (M) RECO-day (ecosystem respiration in daytime), (N) GPP-day (gross primary production in daytime), (O) WUEi-GPP (inherent water use efficiency [equal to GPP-day × VPD/LE-day]). For more detailed information about the variables' units, see Pastorello et al.[25]

RATIOs of >0.55 have warming or cooling effects cannot be determined under the global warming background. To be capable of predicting the effects of ecosystem changes on Ta-day under such daily RATIOs, the change patterns of *r* (the temporal trend of Ta-day) with the daily RATIO should be considered. The curve exhibits a unimodal mode with a vertex at a daily RATIO of ≈0.90 (Figure 3B). When the daily RATIO is < 0.90, *r* increases with increasing daily RATIO, indicating that an increase in the daily RATIO induces warming effects. When the daily RATIO is > 0.90, *r* decreases with increasing daily RATIO, indicating that an increase in the daily RATIO induces cooling effects. Based on the slope changes of the curve in Figure 3B, *r* increases rapidly at daily RATIOs of 0.55–0.75, increases very slowly at daily RATIOs of 0.75–0.90, and exhibits a gradually accelerating decrease at daily RATIOs of 0.90–1, which corresponds to a strong warming effect, a very weak warming effect, and a gradually accelerating cooling effect, respectively.

Contributors to the daily RATIO

Using this approach, the elaboration of the complex BAB feedbacks between ecosystem changes and climate effects is avoided. The climate effects can be quantified from directly measured daily RATIO values if the daily RATIO and climate effects are considered to be interlinked through BAB processes. However, to compare our predictions with those of previous studies, it is still necessary to ascertain what ecosystem change (i.e., alteration of environmental factors) contributes significantly to the daily RATIO, i.e., to establish a common basis on which different approaches can be compared.

Using the random forest model, we screened nine factors (day length, LAI, ΔT [difference in temperature between day and night], P, SWC, CO_2, P/PET [wetness index, P-annual precipitation, and PET-annual potential evapotranspiration], VPD, and WS [wind speed]) that contribute significantly to the daily RATIO (Table S1). The vegetation-driven BAB effects are influenced not only by the ecosystem-specific factors (e.g., LAI, P, SWC, VPD, and WS) but also by the background climate[13,18,19] (e.g., day length, ΔT, and P/PET) and atmospheric CO_2 concentration. In addition to the nine factors we screened, there may be other factors (e.g., plant functional traits, vegetation structure, and albedo) that exert impacts on the daily RATIO and thus on the climate. This is likely why "land surface models show important limitations in reproducing the interplay between vegetation and climate due to an incomplete understanding and model representation of biophysical processes."[6,14]

Figure S6 shows the temporal trend of the LAI worldwide. On average, globally, the LAI has increased over the past two decades. Figure 4 displays the influence of the LAI on the daily RATIO. Although the variation is large, an increase or decrease in the LAI will generally cause the daily RATIO to increase or decrease, respectively, establishing a qualitative connection between the LAI and the daily RATIO.

Comparison of predictions of climate effects in this and previous studies

Taking the LAI changes as a proxy of forest or vegetation changes and based on the established qualitative connection between the LAI and the daily RATIO (Figure 4), Figure 3 predicts the temporal trends of the effects of forest or vegetation changes on the 15 EC variables. Here, we show that the predicted results for two carbon-cycle parameters (NEE and WUE) and the effects of the LAI changes (forest or vegetation changes) on the land surface temperature (Ta-day), and we compare our predictions with those of previous studies.

The NEE and WUE predicted using our approach are highly consistent with those of previous studies.[4,24,26] For example, the temporal trends of the mid-summer NEE-day at 15 EC sites reported by Keenan et al.[24] are consistent with our predictions (Figure 3K). Tang et al.[26] found that the WUE peaks at about 51°N, where the daily RATIO is within 0.80–0.90 and where the WUEi-GPP is highest (Figure 3O).

In addition, we compared our predictions of the effects of the LAI changes on Ta-day with those of land surface models.

In the tropical zone (23°S–23°N), the annual mean daily RATIO of all 22 sites was 0.94, falling within the daily RATIO range of >0.90, in which vegetation increases led to a strong cooling effect, and vice versa.[1,27-31] However, in the tropical drylands (SWC < 10%), the annual mean daily RATIO was 0.91, so the cooling effect was not as strong as that at all of the 22 sites.[9] For the 5 degraded sites (AU-Rob, AU-TTE, AU-Fog, SN-Dhr, and AU-ASM), where the LAI decreased, the

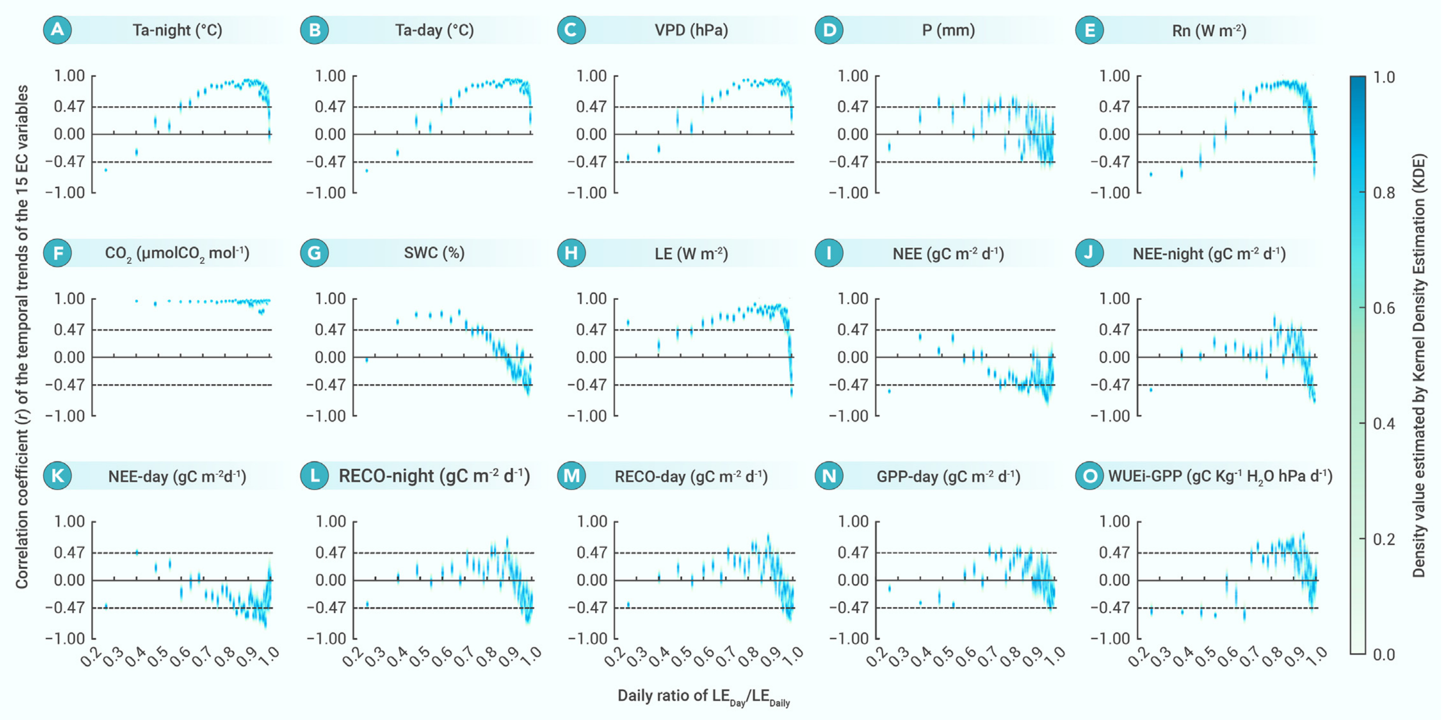

Figure 3. Correlation coefficients (*r*) of the temporal trends of the 15 EC variables from 1997 to 2014 under different daily RATIOs Labels (A)–(O) are consistent with those mentioned in Figures 1 and 2. The distribution of the *r* values is shown by the color scale. A deeper shade of blue corresponds to a denser distribution. Two dashed lines mark the range of the statistical significance, $r > 0.47$ or $r < -0.47$ indicate that the temporal trend of the variable during 1997–2014 is positively or negatively significant, respectively. All 212 sites are included. The number of bootstrap samples is 1,000 (B = 1,000), the number of samples for each bootstrap sample is 4,000 (bootstrap sample size = 4,000), and the least number of unique site names to ensure in each sample is 10 (least unique station requirement = 10).

annual mean daily RATIO was 0.86, so the Ta-day and NEE were higher and lower, respectively, compared with those of the undegraded sites.[5]

In the subtropical zones (23°S–35°S and 23°N–35°N), the annual mean daily RATIO of all 22 sites was 0.91, so vegetation increases generated a weak cooling effect. In the subtropical drylands (SWC < 10%), the annual mean daily RATIO was 0.92, so the cooling effect of the vegetation increases in the subtropical drylands was stronger than that at all 22 sites. The annual mean daily RATIO of the other subtropical sites, excluding the drylands, was 0.89, falling within the daily RATIO range of 0.75–0.90 and approaching the upper limit. Under this condition, a slight change in the vegetation would not affect the Ta-day much, but a large increase in vegetation could cool the land.

In the temperate zones (35°S–60°S and 35°N–60°N), the annual mean daily RATIO of all 148 sites was 0.85, falling within the daily RATIO range of 0.75–0.90, where vegetation changes had minimal effects on the Ta-day, usually a weak warming effect.[1,8,29] The increase in vegetation resulting from the earlier leafing-out in the boreal and northern temperate regions (>50° NS) induced weak warming effects[32] as a result of the annual mean daily RATIO value of 0.81.

In the boreal zones (>60°N and >60°S), the annual mean daily RATIO of all 20 sites was 0.73, falling within the daily RATIO range of <0.75, so vegetation increases led to a strong warming effect, and vice versa.[1,27-31] In the 9 years of 1997–2002, 2004–2005, and 2009, the annual mean daytime temperature and precipitation were −1.7°C and 391 mm (warm-dry), and the annual mean daily RATIO of all 20 sites was 0.75. In the 9 years of 2003, 2006–2008, and 2010–2014, the annual mean daytime temperature and precipitation were −1.9°C and 442 mm (cold-wet), and the annual mean daily RATIO of all 20 sites was 0.72. The sensitivity of the warming effect to a unit increase in vegetation was much weaker in the warm-dry years than in the cold-wet years.[31]

In the arid regions (P/PET < 0.50 and SWC < 10%), the annual mean daily RATIO of all 37 sites was 0.91, and a vegetation increase led to a weak cooling effect.[31] In 2009, the annual mean daytime temperature, precipitation, and mean daily RATIO of all 37 sites were 23.7°C, 399 mm (warm-dry), and 0.92, respectively; while in 2011, they were 23.0°C, 429 mm (cold-wet), and 0.88, respectively. From the warm-dry years to the cold-wet years, the biophysical effects of the regions changed from cooling to warming effects.[31]

The annual mean daily RATIO of the three sites (CN-Dan, CN-Ha2, and CN-HaM) on the Tibetan Plateau was 0.94. Vegetation increases in this region will certainly induce a strong cooling effect, which is different from the situation in boreal zones.[19,33]

During 1997–2014, the annual mean daily RATIO and LAI of the vast regions with latitudes of 60°S to 60°N were 0.87 and 1.73, respectively. An ongoing greener globe (Figure S6) will increase the annual mean daily RATIO and thus will exert cooling effects on land surfaces. This has also been predicted in previous studies.[10,34] In China, the cooling effect emerged during this period[34] as the annual mean daily RATIO of the 10 sites in this region (CN-Cha, Cng, Dan, Din, Du2, Du3, Ha2, HaM, Qia, and Sw2) was 0.90.

The mean daily RATIOs in summer, autumn, and spring in the Northern Hemisphere were 0.95, 0.90, and 0.84, respectively. Seasonal greening will generate strong and weak cooling effects in summer and autumn, respectively, and a weak warming effect in spring.[35]

Su et al.[36] found that, in many forests, the magnitude of biophysical cooling due to an increase in tree cover is greater than that of warming due to a decrease in tree cover. In fact, the annual mean daily RATIO of all forest sites in the vast regions between 40°S and 40°N was 0.90, falling on the border between the two ranges of 0.75–0.90 and >0.90, in which the sensitivity of biophysical cooling to tree cover gain is much stronger than that of biophysical warming to tree cover loss.

In summary, our predictions of the effects of ecosystem changes on surface temperature in different climatic zones (including the Tibetan Plateau) and several unique environments are highly consistent with those of all previous studies. However, our analysis and reasoning does not support the general consensus that cooling or warming effects are induced by enhanced or reduced evapotranspiration.[5,15,35-39] Instead, we conclude that they are controlled by the daily RATIO.

About the approach and its applications

Black box and white box approaches are usually applied to study the effects of ecosystem changes on climate. Black box approaches seldom consider the BAB processes between ecosystem changes and climate effects and concentrate on the statistical relationships between inputs and outputs. Experiments related to the effects of ecosystem warming or disturbance, CO_2 enrichment,

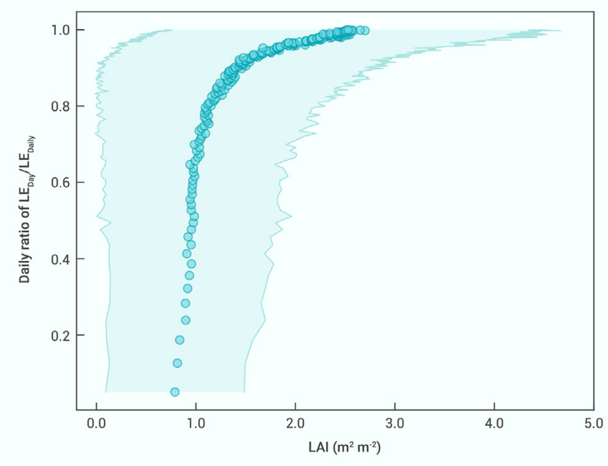

Figure 4. Influences of leaf area index on the daily RATIO The shaded area denotes the standard deviation of the data.

nitrogen addition, and precipitation alteration on climate have often used this approach. In contrast, white box approaches try to clarify the BAB processes so that the output can be determined from the input. Many land surface models can be classified as such approaches. Our approach can be called a fast track approach, which is completely different from black box and white box approaches. We mind the black box but do not intend to open it. Instead, we bypass it with the assistance of a guider located in the black box. The daily RATIO plays the role of the guider, representing the effects of ecosystem changes on the daily RATIO but not the ecosystem changes themselves. That is, the fast track approach starts from the known daily RATIO, not from ecosystem changes as the black box and white box approaches do. Based on the daily RATIO effect of the ecosystem changes produced in the black box, we can infer the climate effects of the ecosystem changes that occur in the same black box.

The theory of the fast track approach is robust, and the daily RATIO seems to be a universal guider, making it capable of capturing the ecosystem's complexity and diversity. If the daily RATIOs of an ecosystem at any spatiotemporal scale are known, our approach can be used to evaluate and predict the corresponding climate effects of ecosystem changes. However, our approach still requires further study. First, the daily RATIO may not be the best guider. We hypothesize that the ratio of transpiration to evaporation would perform better than the daily RATIO in our approach, but we did not use it in this paper due to the difficulty of separating transpiration and evaporation from evapotranspiration.[40-44] Second, the connections, even the qualitative connections, between the daily RATIO and many ecosystem properties (e.g., plant functional traits, vegetation structure, and albedo) and ecosystem managements (artificially induced ecosystem changes) have not been established. Consequently, it is difficult to compare the fast track, black box, and white box approaches because the latter two are usually based directly on ecosystem properties and managements. Naturally, the applications of the fast track approach in the formulation of policy and management planning are also limited because routine observations on global ecosystems do not include the daily evapotranspiration, even though it is becoming easy to measure with the development of EC technology and remote sensing.

Nevertheless, by taking LAI changes as a proxy for forest or vegetation changes and based on the qualitative connection between the daily RATIO and LAI (Figure 4), in this study we successfully predicted the effects of forest or vegetation changes on the land surface temperature across the globe, and our results are consistent with those of 33 studies.[1,4,5,8-10,15,19,24,26-39] We also demonstrated that our approach has a powerful evaluation capability and is superior to land surface models in this aspect, providing an innovative method for investigating the climate effects of complex system alterations. In view of this, in addition to the continuation of remote sensing and EC observations, we suggest that high-spatial-resolution observations of the daily (daytime and nighttime) evapotranspiration should be conducted globally. This work will be beneficial to optimizing terrestrial ecosystem management to mitigate global climate change and improve ecosystem health.

MATERIALS AND METHODS

Database construction

We downloaded both the high-frequency (30 min) dataset and the daily dataset from the published database[25] (https://fluxnet.org/data/fluxnet2015-dataset/). The LAI data were obtained from GLOBMAP v.3 (https://zenodo.org/record/4700264), which fuses Advanced Very High Resolution Radiometer (AVHRR) and Moderate Resolution Imaging Spectroradiometer (MODIS) data to provide long-term and continuous products.[45] The annual precipitation (annual potential evapotranspiration [P/PET] wetness index) was calculated according to the method of Zhou et al.[46]

Daily RATIO

The daily RATIO and day length (or night length) were calculated from the high-frequency (30 min) dataset. We believe that, under any extreme geographic background, vegetation coverage, and instantaneous microclimate, the daily RATIO will not exceed the range of (0, 1); therefore, 23.4% of the calculated daily RATIOs were discarded because they exceeded this range.

Figure S7 shows the daily RATIO values of the various EC sites, vegetation types, latitudes, and months, as well as in-year processes, of the daily RATIO for evergreen broadleaved, deciduous broadleaved, evergreen needle-leaved, and deciduous needle-leaved forests.

Coupling EC variables with the daily RATIO

The daily RATIO range was divided into many sub-intervals (200 sub-intervals). The random forest model and polynomial model were used to quantify the coupling relationships between the daily RATIO and the different EC variables across different ecosystem types and timescales.

Using the daily RATIO to map EC observations from one place to other places

Based on the quantitative coupling relationships established for one place, the random forest model and polynomial model were used to estimate the EC variables in other places according to the daily RATIOs in these other places.

Temporal trends of EC variables for different daily RATIOs

The daily RATIO range was divided into 50 sub-intervals. For each sub-interval, bootstrap sampling was used to sample various combinations of EC observation sites. For each combination of sites, the linear model was used to regress the relationships between the EC variables and years. The correlation coefficients r ($r = \pm\sqrt{R^2}$) of these regressed relationships were plotted against the median of the sub-intervals of the daily RATIO. The effects of ecosystem changes on the EC variables are implied in the curves of r versus the daily RATIO.

DATA AND CODE AVAILABILITY

All data of this study are publicly available at https://figshare.com/s/f0b894406be788267a5a.

REFERENCES

1. Bala, G., Caldeira, K., Wickett, M., et al. (2007). Combined climate and carbon-cycle effects of large-scale deforestation. Proc. Natl. Acad. Sci. USA ***104***(16): 6550–6555. https://doi.org/10.1073/pnas.0608998104.
2. Zhu, P., Zhuang, Q., Eva, J., et al. (2016). Importance of biophysical effects on climate warming mitigation potential of biofuel crops over the conterminous United States. GCB Bioenergy ***9***(3): 577–590. https://doi.org/10.1111/gcbb.12370.
3. Windisch, M.G., Davin, E.L., et al. (2021). Prioritizing forestation based on biogeochemical and local biogeophysical impacts. Nat. Clim. Change ***11***(10): 867–871. https://doi.org/10.1038/s41558-021-01161-z.
4. Tong, X., Xiao, J., Liu, P., et al. (2023). Carbon exchange of forest plantations: global patterns and biophysical drivers. Agric. For. Meteorol. ***336***: 109379. https://doi.org/10.1016/j.agrformet.2023.109379.
5. Zhu, L., Li, W., Ciais, P., et al. (2023). Comparable biophysical and biogeochemical feedbacks on warming from tropical moist forest degradation. Nat. Geosci. ***16***(3): 244–249. https://doi.org/10.1038/s41561-023-01137-y.
6. Duveiller, G., Forzieri, G., Robertson, E., et al. (2018). Biophysics and vegetation cover change: a process-based evaluation framework for confronting land surface models with satellite observations. Earth Syst. Sci. Data ***10***(3): 1265–1279. https://doi.org/10.5194/essd-10-1265-2018.
7. Zhou, D., Xiao, J., Frolking, S., et al. (2021). Croplands intensify regional and global warming according to satellite observations. Remote Sens. Environ. ***264***: 112585. https://doi.org/10.1016/j.rse.2021.112585.
8. Luyssaert, S., Jammet, M., Stoy, P.C., et al. (2014). Land management and land-cover change have impacts of similar magnitude on surface temperature. Nat. Clim. Change ***4***(5): 389–393. https://doi.org/10.1038/nclimate2196.

9. Feldman, A.F., Short Gianotti, D.J., Dong, J., et al. (2023). Tropical surface temperature response to vegetation cover changes and the role of drylands. Global Change Biol. ***29***(1): 110–125. https://doi.org/10.1111/gcb.16455.
10. Alkama, R., Forzieri, G., Duveiller, G., et al. (2022). Vegetation-based climate mitigation in a warmer and greener World. Nat. Commun. ***13***(1): 606. https://doi.org/10.1038/s41467-022-28305-9.
11. He, N., Yan, P., Liu, C., et al. (2023). Predicting ecosystem productivity based on plant community traits. Trends Plant Sci. ***28***(1): 43–53. https://doi.org/10.1016/j.tplants.2022.08.015.
12. Chen, Z., Wang, W., Cescatti, A., et al. (2023). Climate-driven vegetation greening further reduces water availability in drylands. Global Change Biol. ***29***(6): 1628–1647. https://doi.org/10.1111/gcb.16561.
13. Forzieri, G., Miralles, D.G., Ciais, P., et al. (2020). Increased control of vegetation on global terrestrial energy fluxes. Nat. Clim. Change ***10***(4): 356–362. https://doi.org/10.1038/s41558-020-0717-0.
14. Forzieri, G., Duveiller, G., Georgievski, G., et al. (2018). Evaluating the Interplay Between Biophysical Processes and Leaf Area Changes in Land Surface Models. J. Adv. Model. Earth Syst. ***10***(5): 1102–1126. https://doi.org/10.1002/2018MS001284.
15. Zeng, Z., Piao, S., Li, L.Z.X., et al. (2017). Climate mitigation from vegetation biophysical feedbacks during the past three decades. Nat. Clim. Change ***7***(6): 432–436. https://doi.org/10.1038/nclimate3299.
16. Qin, S., Fan, Y., Li, S., et al. (2023). Partitioning of available energy in canopy and soil surface in croplands with different irrigation methods. Agric. Water Manag. ***288***: 108475. https://doi.org/10.1016/j.agwat.2023.108475.
17. Li, Y., Piao, S., Chen, A., et al. (2020). Local and teleconnected temperature effects of afforestation and vegetation greening in China. Natl. Sci. Rev. ***7***(5): 897–912. https://doi.org/10.1093/nsr/nwz132.
18. Pitman, A.J., Avila, F.B., Abramowitz, G., et al. (2011). Importance of background climate in determining impact of land-cover change on regional climate. Nat. Clim. Change ***1***(9): 472–475. https://doi.org/10.1038/nclimate1294.
19. Shen, P., and Zhao, S. (2021). 1/4 to 1/3 of observed warming trends in China from 1980 to 2015 are attributed to land use changes. Clim. Change ***164***(3): 59. https://doi.org/10.1007/s10584-021-03045-9.
20. Jarvis, P.G. (1976). The interpretation of the variations in leaf water potential and stomatal conductance found in canopies in the field. Philos. Trans. R. Soc. London, Ser. A B ***273***(927): 593–610.
21. Monteith, J.L. (1965). Evaporation and environment. Symp. Soc. Exp. Biol. ***19***: 205–234.
22. Katul, G.G., Oren, R., Manzoni, S., et al. (2012). Evapotranspiration: a process driving mass transport and energy exchange in the soil-plant-atmosphere-climate system. Rev. Geophys. ***50***(3). https://doi.org/10.1029/2011RG000366.
23. Wang, K., and Dickinson, R.E. (2012). A review of global terrestrial evapotranspiration: Observation, modeling, climatology, and climatic variability. Rev. Geophys. ***50***(2). https://doi.org/10.1029/2011rg000373.
24. Keenan, T.F., Hollinger, D.Y., Bohrer, G., et al. (2013). Increase in forest water-use efficiency as atmospheric carbon dioxide concentrations rise. Nature ***499***(7458): 324–327. https://doi.org/10.1038/nature12291.
25. Pastorello, G., Trotta, C., Canfora, E., et al. (2020). The FLUXNET2015 dataset and the ONEFlux processing pipeline for eddy covariance data. Sci. Data ***7***(1): 225. https://doi.org/10.1038/s41597-020-0534-3.
26. Tang, X., Li, H., Desai, A.R., et al. (2014). How is water-use efficiency of terrestrial ecosystems distributed and changing on Earth? Sci. Rep. ***4***(1): 7483. https://doi.org/10.1038/srep07483.
27. Bonan, G.B., Pollard, D., and Thompson, S.L. (1992). Effects of boreal forest vegetation on global climate. Nature ***359***(6397): 716–718. https://doi.org/10.1038/359716a0.
28. Betts, R.A. (2000). Offset of the potential carbon sink from boreal forestation by decreases in surface albedo. Nature ***408***(6809): 187–190. https://doi.org/10.1038/35041545.
29. Bonan, G.B. (2008). Forests and climate change: forcings, feedbacks, and the climate benefits of forests. Science ***320***(5882): 1444–1449. https://doi.org/10.1126/science.1155121.
30. Lee, X., Goulden, M.L., Hollinger, D.Y., et al. (2011). Observed increase in local cooling effect of deforestation at higher latitudes. Nature ***479***(7373): 384–387. https://doi.org/10.1038/nature10588.
31. Forzieri, G., Alkama, R., Miralles, D.G., et al. (2017). Satellites reveal contrasting responses of regional climate to the widespread greening of Earth. Science ***356***(6343): 1180–1184. https://doi.org/10.1126/science.aal1727.
32. Xu, X., Riley, W.J., Koven, C.D., et al. (2020). Earlier leaf-out warms air in the north. Nat. Clim. Change ***10***(4): 370–375. https://doi.org/10.1038/s41558-020-0713-4.
33. Liu, W., Zhou, Y., Dong, J., et al. (2023). Cooling effects of increased green fodder area on native grassland in the northeastern Tibetan Plateau. Environ. Res. Lett. ***18***(6): 064006. https://doi.org/10.1088/1748-9326/acc9d3.
34. Li, Y., Li, Z.L., Wu, H., et al. (2023). Biophysical impacts of earth greening can substantially mitigate regional land surface temperature warming. Nat. Commun. ***14***(1): 121. https://doi.org/10.1038/s41467-023-35799-4.
35. Lian, X., Jeong, S., Park, C.E., et al. (2022). Biophysical impacts of northern vegetation changes on seasonal warming patterns. Nat. Commun. ***13***(1): 3925. https://doi.org/10.1038/s41467-022-31671-z.
36. Su, Y., Zhang, C., Ciais, P., et al. (2023). Asymmetric influence of forest cover gain and loss on land surface temperature. Nat. Clim. Change ***13***(8): 823–831. https://doi.org/10.1038/s41558-023-01757-7.
37. Shen, M., Piao, S., Jeong, S.J., et al. (2015). Evaporative cooling over the Tibetan Plateau induced by vegetation growth. Proc. Natl. Acad. Sci. USA ***112***(30): 9299–9304. https://doi.org/10.1073/pnas.1504418112.
38. Zeng, Z., Wang, D., Yang, L., et al. (2020). Deforestation-induced warming over tropical mountain regions regulated by elevation. Nat. Geosci. ***14***(1): 23–29. https://doi.org/10.1038/s41561-020-00666-0.
39. Li, Z., Ciais, P., Wright, J.S., et al. (2023). Increased precipitation over land due to climate feedback of large-scale bioenergy cultivation. Nat. Commun. ***14***(1): 4096. https://doi.org/10.1038/s41467-023-39803-9.
40. Wilson, K.B., Hanson, P.J., and Baldocchi, D.D. (2000). Factors controlling evaporation and energy partitioning beneath a deciduous forest over an annual cycle. Agric. For. Meteorol. ***102***(2-3): 83–103. https://doi.org/10.1016/S0168-1923(00)00124-6.
41. Wilson, K.B., Hanson, P.J., Mulholland, P.J., et al. (2001). A comparison of methods for determining forest evapotranspiration and its components: sap-flow, soil water budget, eddy covariance and catchment water balance. Agric. For. Meteorol. ***106***(2): 153–168. https://doi.org/10.1016/S0168-1923(00)00199-4.
42. Oishi, A.C., Oren, R., and Stoy, P.C. (2008). Estimating components of forest evapotranspiration: a footprint approach for scaling sap flux measurements. Agric. For. Meteorol. ***148***(11): 1719–1732. https://doi.org/10.1016/j.agrformet.2008.06.013.
43. Liu, X., Li, Y., Chen, X., et al. (2015). Partitioning evapotranspiration in an intact forested watershed in southern China. Ecohydrology ***8***(6): 1037–1047. https://doi.org/10.1002/eco.1561.
44. Scott, R.L., and Biederman, J.A. (2017). Partitioning evapotranspiration using long-term carbon dioxide and water vapor fluxes. Geophys. Res. Lett. ***44***(13): 6833–6840. https://doi.org/10.1002/2017GL074324.
45. Liu, Y., Liu, R., and Chen, J.M. (2012). Retrospective retrieval of long-term consistent global leaf area index (1981–2011) from combined AVHRR and MODIS data. J. Geophys. Res. ***117***(G4): G04003. https://doi.org/10.1029/2012JG002084.
46. Zhou, G., Wei, X., Chen, X., et al. (2015). Global pattern for the effect of climate and land cover on water yield. Nat. Commun. ***6***(1): 5918. https://doi.org/10.1038/ncomms6918.

ACKNOWLEDGMENTS

This work was supported by the National Natural Science Foundation of China (nos. 42130506 and 42071031), and the Special Technology Innovation Fund of Carbon Peak and Carbon Neutrality in Jiangsu Province (no. BK20231515).

AUTHOR CONTRIBUTIONS

G.Z. analyzed the dataset, designed the study, and wrote the paper. L.H. and L.L. assimilated the dataset, carried out the calculations, and made some drawings. W.C., X.W., and X.X. carried out the calculations and made some drawings.

DECLARATION OF INTERESTS

The authors declare no competing interests.

SUPPLEMENTAL INFORMATION

It can be found online at https://doi.org/10.1016/j.xinn.2024.100733.

LEAD CONTACT WEBSITE

https://faculty.nuist.edu.cn/zhouguoyi/zh_CN/index.htm.

The Innovation, Volume *6*

Supplemental Information

Climate effects of ecosystem change converge according to the ratio of the daytime to daily vapor flux

Langqin Hua, Lin Li, Wenjing Chen, Xuemeng Wang, Xin Xiong, and Guoyi Zhou

Supplemental Information

Climate effects of ecosystem change converge according to the ratio of daytime

to daily vapor flux

DOI:

Langqin Hua, Lin Li, Wenjing Chen, Xuemeng Wang, Xin Xiong, and Guoyi Zhou

Table of Contents

Figures S1 to S7

Tables S1

REFERENCES

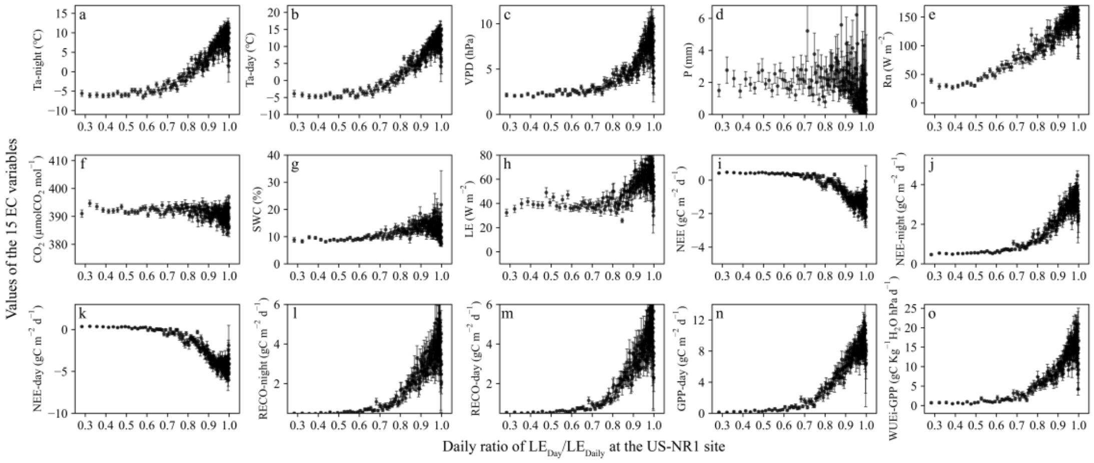

Figure S1. Changes in eddy-covariance variables with the daily ratio of the daytime to daily vapor flux (the daily RATIO) at the US-NR1 site. The variables include a) Ta-night - air temperature in the non-photosynthetic period, b) Ta-day - air temperature in the photosynthetic period, c) VPD - vapor pressure deficit, d) P - daily precipitation, e) Rn - net radiation, f) CO_2 - atmospheric CO_2 concentration, g) SWC - soil water content, h) LE - vapor flux (evapotranspiration), i) NEE - net carbon exchange of ecosystem, j) NEE-night - net carbon exchange of ecosystem at night, k) NEE-day - net carbon exchange of ecosystem in daytime, l) RECO-night - ecosystem respiration at night, m) RECO-day - ecosystem respiration in daytime, n) GPP-day - gross primary production in daytime, and o) WUEi-GPP - inherent water use efficiency (equal to GPP-day*VPD/LE-day). See Pastorello et al.[1] for more detailed information about the site and variables.

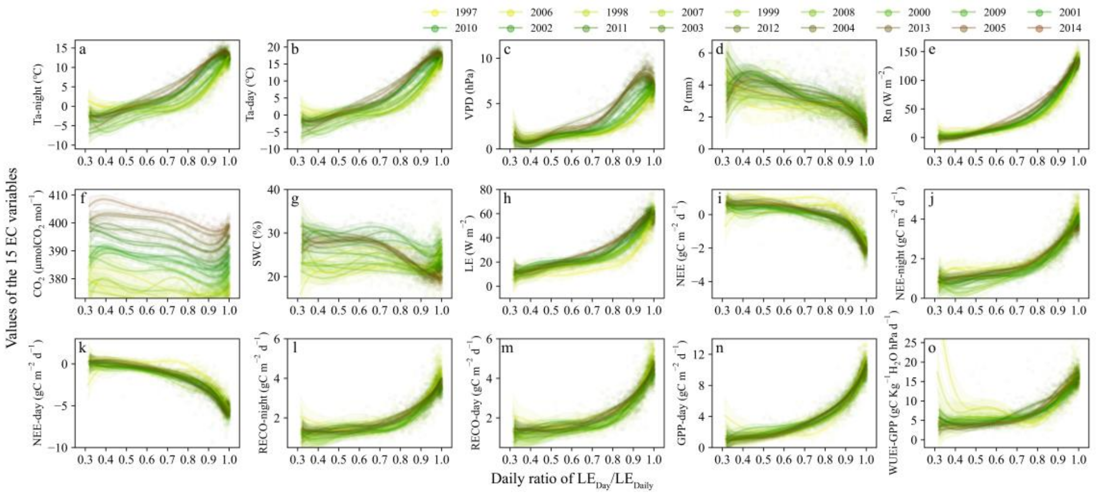

Figure S2. Variations of eddy covariance variables with the daily ratio of the daytime to daily vapor flux (the daily RATIO) for each year during 1997-2014. The fitted curves are all statistically significant (p<0.05). The variables include a) Ta-night - air temperature in the non-photosynthetic period, b) Ta-day - air temperature in the photosynthetic period, c) VPD - vapor pressure deficit, d) P - daily precipitation, e) Rn - net radiation, f) CO_2 - atmospheric CO_2 concentration, g) SWC - soil water content, h) LE - vapor flux (evapotranspiration), i) NEE - net carbon exchange of ecosystem, j) NEE-night - net carbon exchange of ecosystem at night, k) NEE-day - net carbon exchange of ecosystem in daytime, l) RECO-night - ecosystem respiration at night, m) RECO-day - ecosystem respiration in daytime, n) GPP-day - gross primary production in daytime, and o) WUEi-GPP - inherent water use efficiency (equal to GPP-day*VPD/LE-day). See Pastorello et al.[1] for more detailed information about the site and variables.

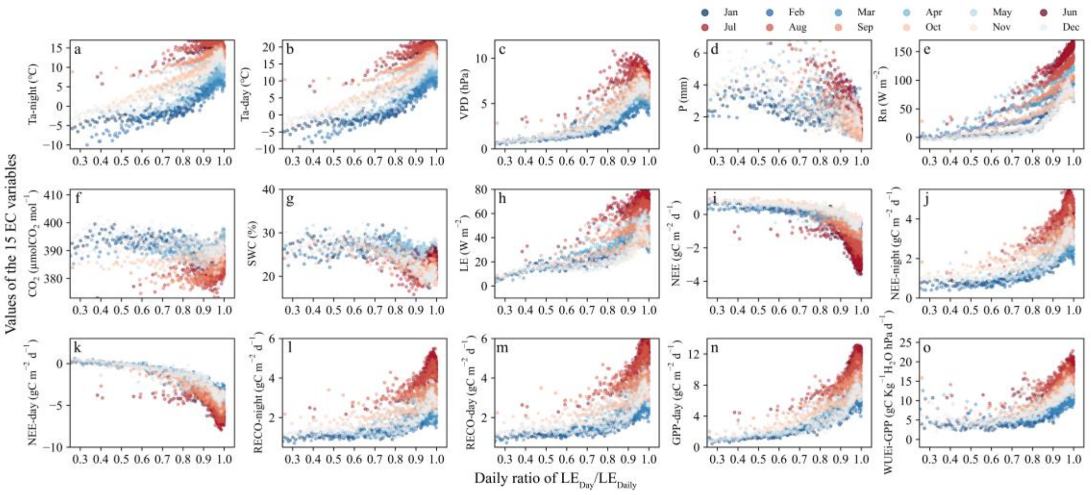

Figure S3. Monthly eddy covariance variables with the daily ratio of the daytime to daily vapor flux (the daily RATIO) during 1997-2014. The variables include a) Ta-night - air temperature in the non-photosynthetic period, b) Ta-day - air temperature in the photosynthetic period, c) VPD - vapor pressure deficit, d) P - daily precipitation, e) Rn - net radiation, f) CO_2 - atmospheric CO_2 concentration, g) SWC - soil water content, h) LE - vapor flux (evapotranspiration), i) NEE - net carbon exchange of ecosystem, j) NEE-night - net carbon exchange of ecosystem at night, k) NEE-day - net carbon exchange of ecosystem in daytime, l) RECO-night - ecosystem respiration at night, m) RECO-day - ecosystem respiration in daytime, n) GPP-day - gross primary production in daytime, and o) WUEi-GPP - inherent water use efficiency (equal to GPP-day*VPD/LE-day). See Pastorello et al.[1] for more detailed information about the site and variables.

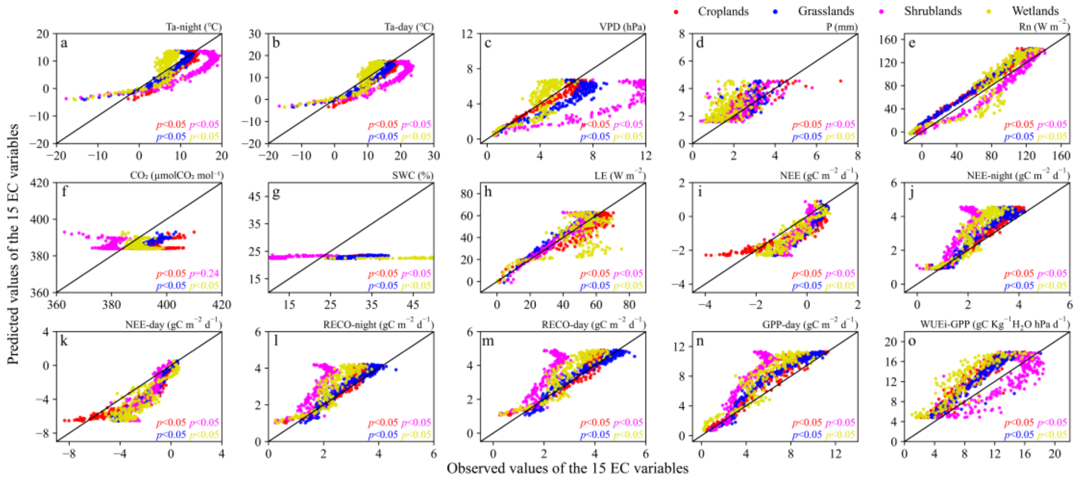

Figure S4. Comparison of predicted and observed daily values of the 15 variables for cropland, grassland, shrubland and wetland. The observed values are the measurements in the four land cover types. The predicted values are based on the measured daily RATIOs in the four land cover types and the regressed relationship between EC variables and daily RATIO in forest. *p* values indicate the significance of the relationships between predicted and observed values. Shrubland in the figure is the combination of shrubland and savanna in Pastorello et al.[1]

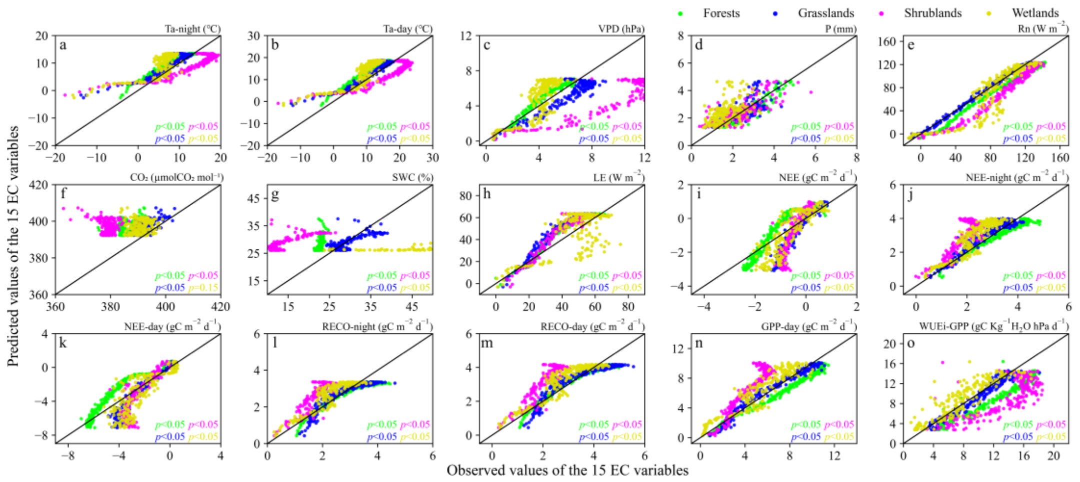

Figure S5. Comparison of predicted and observed daily values of the 15 variables for forest, grassland, shrubland and wetland. The observed values are the measurements in the four land cover types. The predicted values are based on the measured daily RATIOs in the four land cover types and the regressed relationship between EC variables and daily RATIO in croplands. *p* values indicate the significance of the relationships between predicted and observed values. Shrubland in the figure is the combination of shrubland and savanna in Pastorello et al.[1]

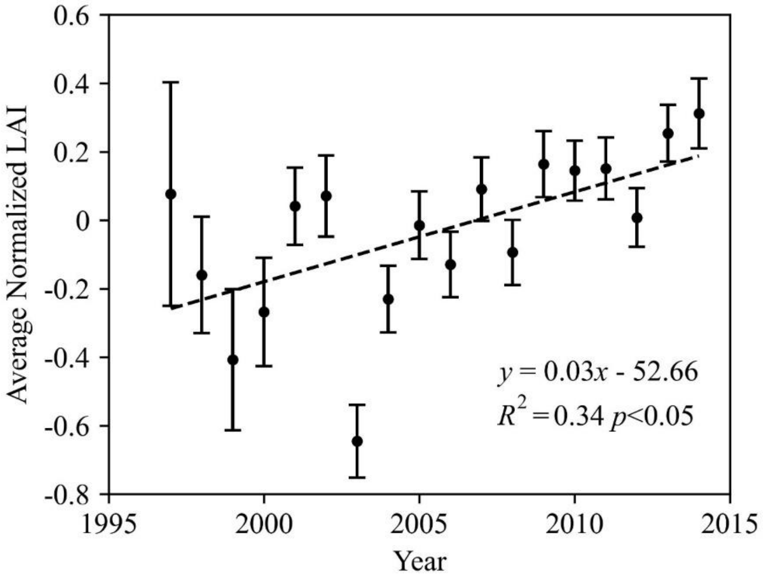

Figure S6. The tendency of global leaf area index (LAI) from 1997-2014. The LAI data were standardized using z-score normalization for each site. Error bars indicate the standard error of the mean.

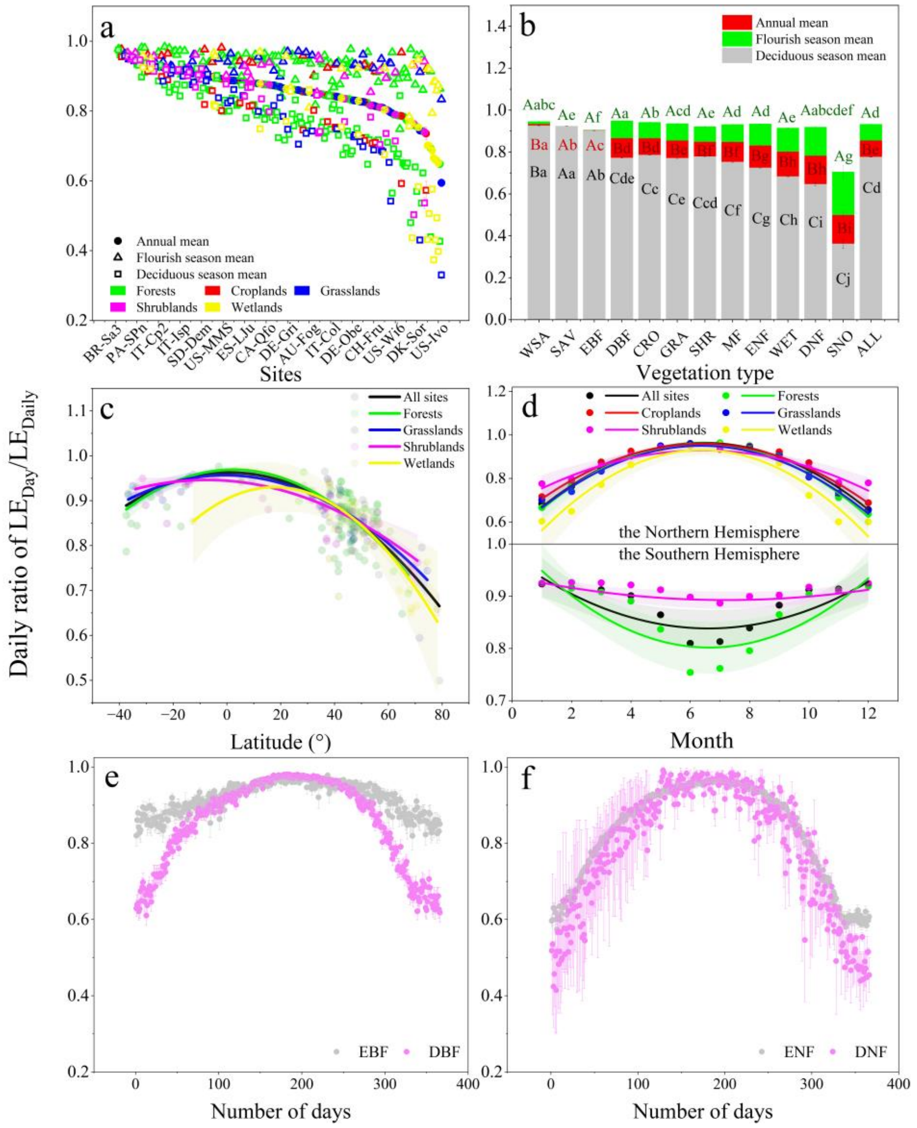

Figure S7. Daily ratio of the daytime to daily vapor flux (the daily RATIO). Averaged according to **a**) eddy covariance (EC) sites, **b**) vegetation types, **c**) latitudes and **d**) months. In-year process of daily RATIO for **e**) evergreen broadleaved forest (grey color) and deciduous broadleaved forest (pink color), **f**) evergreen needle-leaved forest (grey color) and deciduous needle-leaved forest (pink color). BR-Sa3, …, NO-Blv - names of the 212 sites, WSA - woody savanna, SAV - savanna, EBF - evergreen broadleaved forest, DBF - deciduous broadleaved forest, CRO - cropland, GRA - grassland, SHR - shrubland, MF - mixed forest, ENF - evergreen needle-leaved forest, WET - wetlands, DNF - deciduous needle-leaved forest, SNO - snow and ice cover.

Error bars indicate standard errors of the means. Different capital letters indicate a significant ($p<0.05$) difference between annual, flourish season and deciduous season mean daily RATIO, while the different lowercase letters indicate a significant ($p<0.05$) difference between different vegetation types. Flourish season refers to April to September in the Northern Hemisphere and October to March of the following year in the Southern Hemisphere, while deciduous season refers to the other six months of the year.

Table S1 Contributions of the nine significant contributors to daily RATIO in accordance with climate regions and ecosystem types

	All	Tropic	Subtropics	Temperate	Frigid	Forest	Crop	Grass	Shrubsav	Wet
R^2	0.62	0.59	0.52	0.60	0.85	0.63	0.63	0.66	0.55	0.65
n	258180	24568	54166	173828	5618	142980	26444	50603	37165	988
LAI	6.49	22.25	8.16	6.56	2.14	6.51	5.95	6.35	10.31	16.61
ΔT	11.20	17.49	21.76	12.49	2.93	9.28	17.54	13.93	17.09	20.62
Day Length	43.73	2.73	10.64	44.61	81.12	45.03	42.05	47.65	26.36	4.13
P	4.51	5.43	6.92	4.86	0.96	4.77	4.57	3.16	5.11	2.45
SWC	6.05	15.94	9.18	6.18	2.42	5.77	6.08	6.51	9.03	12.38
CO_2	5.73	8.17	8.64	5.87	3.35	5.67	5.76	5.39	7.47	6.30
P/PET	5.94	6.80	7.36	6.07	1.82	5.42	6.09	4.87	5.69	10.94
VPD	9.32	8.84	16.38	6.25	3.09	9.80	6.46	5.93	9.67	19.90
WS	7.03	12.35	10.96	7.11	2.17	7.75	5.50	6.21	9.27	6.67

Note: LAI - leaf area index, ΔT - difference in temperature between day and night, Day Length, P - daily precipitation, SWC - soil water content, CO_2 - atmospheric CO_2 concentration, P/PET - wetness index (P - annual precipitation, PET - annual potential evapotranspiration), VPD - vapor pressure deficit, WS - wind speed. Forest - forest land, Crop - cropland, Grass - grassland, Shrubsav - collection of shrubland, savanna and woody savanna, Wet - wetland.

REFERENCES

1. Pastorello, G., Trotta, C., Canfora, E., et al. (2020). The FLUXNET2015 dataset and the ONEFlux processing pipeline for eddy covariance data. Sci. Data ***7***(1): 225. DOI: https://doi.org/10.1038/s41597-020-0534-3.
